# Differential changes to D1 and D2 medium spiny neurons in the 12-month-old Q175^+/-^ mouse model of Huntington’s Disease

**DOI:** 10.1371/journal.pone.0200626

**Published:** 2018-08-17

**Authors:** Joseph W. Goodliffe, Hanbing Song, Anastasia Rubakovic, Wayne Chang, Maria Medalla, Christina M. Weaver, Jennifer I. Luebke

**Affiliations:** 1 Department of Anatomy & Neurobiology, Boston University School of Medicine, Boston, Massachusetts; 2 Department of Mathematics and Computer Science, Franklin & Marshall College, Lancaster, Pennsylvania; University of Alabama at Birmingham, UNITED STATES

## Abstract

Huntington’s Disease (HD) is an autosomal dominant, progressive neurodegenerative disorder caused by deleterious expansion of CAG repeats in the *Huntingtin* gene and production of neurotoxic mutant Huntingtin protein (mHTT). The key pathological feature of HD is a profound degeneration of the striatum and a loss of cortical volume. The initial loss of indirect pathway (D2) medium spiny neuron (MSN) projections in early stages of HD, followed by a loss of direct pathway (D1) projections in advanced stages has important implications for the trajectory of motor and cognitive dysfunction in HD, but is not yet understood. Mouse models of HD have yielded important information on the effects and mechanisms of mHTT toxicity; however, whether these models recapitulate differential vulnerability of D1 vs. D2 MSNs is unknown. Here, we employed 12-month-old Q175^+/-^ x D2-eGFP mice to examine the detailed structural and functional properties of D1 vs. D2 MSNs. While both D1 and D2 MSNs exhibited increased input resistance, depolarized resting membrane potentials and action potential threshold, only D1 MSNs showed reduced rheobase, action potential amplitude and frequency of spontaneous excitatory postsynaptic currents. Furthermore, D1 but not D2 MSNs showed marked proliferative changes to their dendritic arbors and reductions in spine density. Immunohistochemical assessment showed no loss of glutamatergic afferent inputs from cortical and subcortical sources onto identified D1 and D2 MSNs. Computational models constrained by empirical data predict that the increased dendritic complexity in Q175^+/-^ D1 MSNs likely leads to greater dendritic filtering and attenuation of signals propagating to the soma from the dendrites. Together these findings reveal that, by twelve months, D1 and D2 MSNs exhibit distinctive responses to the presence of mHTT in this important mouse model of HD. This further highlights the need to incorporate findings from D1 and D2 MSNs independently in the context of HD models.

## Introduction

Huntington’s Disease (HD) is a progressive neurodegenerative disorder affecting cognitive, psychiatric, and motor functions [1,2]. While the genetic locus of this autosomal disorder has been known since 1993 [3], no effective disease modifying treatments have been developed to date [4,5] (but see [6,7]). The etiological basis of HD is the deleterious expansion of polyglutamine encoding CAG repeats in Exon 1 of the *Huntingtin* (HTT) gene [3] leading to the ubiquitous expression of neurotoxic mutant Huntingtin (mHTT) and extensive degeneration of neurons in the cortex, thalamus, and most prominently the striatum [8–10]. Medium spiny neurons (MSNs) comprise ~90% of the neurons in the striatum and are the recipients of excitatory inputs from the cortex and thalamus [11]. Within the MSN population, two subtypes–D1 and D2– are distinguishable by the expression of the D1 or D2 dopamine (Drd1 and Drd2) receptors, and by their contribution to the direct and indirect pathways, respectively [12–15]. Evidence from post-mortem HD brain has revealed an initial preferential loss of D2 MSN (enkephalin immunopositive) projections to striatal targets in early stages of the disease, while in later stages D1 MSN (substance P immunopositive) projections undergo marked declines [16–21]. Furthermore, longitudinal PET imaging in HD gene carriers reveals a greater initial loss of D2 compared to D1 receptor binding in the striatum [22,23]. These sequential changes in indirect and direct pathway projections to target structures are widely believed to contribute to the biphasic trajectory of motor dysfunction in HD, in which initial disruption of the indirect pathway results in uncontrolled choreiform movements while later in the disease the additional loss of the direct pathway leads to bradykinesia.

Murine genetic models of HD such as the BACHD, R6/2, YAC128 and Q175 have enabled the interrogation of mechanisms underlying the progression of mHTT-related pathological phenotypes in striatal MSNs. Pathological changes to the electrophysiological properties of unidentified MSNs in symptomatic R6/2 [24–26] and Q175 [27–29] mice include higher input resistance (Rn), reduced rheobase (i.e. increased intrinsic excitability) and reduced excitatory postsynaptic current (EPSC) frequency. Furthermore, a reduction in the density of dendritic spines on MSNs has been reported in BACHD [30], R6/2 [24], and Q175 [28] mouse models. These studies revealed functionally important changes to MSN excitability in the presence of mHTT but have not provided insight into the question of differential changes to D1 and D2 MSNs and their projections in HD model mice.

The generation of mice expressing fluorescent proteins under control of Drd1 or Drd2 promoters crossed with HD models has made it possible to determine whether there are selective and differential effects of mHTT on D1 vs. D2 MSN subtypes. Andre et al. [31] employed this approach in the YAC128 and BACHD models to demonstrate that mean spontaneous excitatory synaptic current (sEPSC) frequency in D1 MSNs was increased from HD mice at a younger age (1.5 months) and reduced at 12 months. By contrast, sEPSC frequency did not differ between HD and WT D2 MSNs at either age. In a different study, using immuno-electron microscopy, Deng and coworkers [32] demonstrated that corticostriatal inputs to D1+ dendritic spines (identified with Vglut1 and D1 immunoreactivity, respectively) are lost in the striatal neuropil of 1-year old Q140 model mice while these inputs to D1- (presumptive D2) spines are spared [32]. By contrast, thalamostriatal (Vglut2+) inputs to both D1 and D2 MSNs are reduced at both 4 and 12 months of age [32]. These studies suggest that the progressive alterations in projections from first D2 and then D1 MSNs seen in human HD does not translate to a similar trajectory of selective vulnerability of MSNs and their projections in HD model mice. Indeed, there is scant data relevant to the question of whether changes predicted by human HD pathological progression in striatal projections are replicated in mouse models.

Here, we address this question using the zQ175neo+^+/-^ and Q175neo-^+/-^ mouse models (hereafter “Q175^+/-^” refers to data from both models combined). The Q175 knock-in (KI) mouse model carries a chimeric human/mouse *HTT* exon 1 carrying an expanded CAG repeat and the human polyproline region within the native murine *htt* gene. Thus, Q175^+/-^ mice have mHTT/HTT construct validity as regards the human disease, recapitulate molecular phenotypes seen in HD pathology, and have clear motoric and cognitive deficits [33]. We employed whole-cell patch clamp recordings and biocytin filling of MSNs in *in vitro* slices of the striatum from 12-month-old (symptomatic) Q175^+/-^ mice to assess whether the structure and function of D1 and D2 MSNs are differentially affected. In addition, we used immunohistochemistry on slices containing biocytin-filled neurons to determine whether there are altered putative inputs to these neurons from cortical (Vglut1+) and subcortical (Vglut2+) glutamatergic afferents. Empirical data were then used to generate computational models of D1 and D2 MSNs to predict functional effects of mHTT-related changes in MSNs from mutant mice. We expected 12-month-old Q175^+/-^ mice to show significant alterations to both D1 and D2 MSNs, as human literature suggests both sub-types to be affected by mHTT in advanced stages of HD. However, our results indicate that while the structural and functional properties of D1 MSNs were significantly altered, D2 MSNs were comparatively less affected in the dorsolateral striatum of aged Q175^+/-^ mice.

## Materials and methods

### Experimental subjects

Mice were obtained from CHDI Foundation at ~11 months-of-age and all experiments were performed on animals at 12 months-of-age. All animals were handled according to animal care guidelines from the NIH *Guide for the Care and Use of Laboratory Animals* and *the U*.*S*. *Public Health Service Policy on Humane Care and Use of Laboratory Animals* and research procedures were approved by the Institutional Animal Care and Use Committee at Boston University School of Medicine. We employed both *z*Q175 neo+^+/-^;Drd2-eGFP^*hemi*^ (CHDI-81008006) and zQ175 neo-+/-;Drd2-eGFP^*hemi*^ (CHDI-81008019) male and female mice maintained on a C57BL/6J background strain in these studies. These mice differ only in the presence or absence of a floxed neomycin resistance cassette upstream of the Htt gene locus. Removal of the neomycin cassette results in an increase of about 10–15% in CNS mHTT expression, but otherwise behavioral and molecular phenotypes are largely indistinguishable between the different lines (CHDI Foundation communication). In our study, the 95% confidence intervals of the median of electrophysiological and anatomical data between these models and sexes were constructed [34] and overlapped in each case. Since this suggested there were no significant differences in the models and sexes, data were pooled for statistical analyses. A total of 16 WT (WT; 7 female, 9 male) 9 *z*Q175 neo+^+/-^;Drd2-eGFP^*hemi*^ (4 females, 5 males; mean ± SD CAG length 186 ± 1.2), and 9 zQ175 neo-^+/-^;Drd2-eGFP^*hemi*^ (6 males, 3 females; mean ± SD CAG length 188 ± 2) mice were studied.

### Slice preparation

Mice were anesthetized with isoflurane and rapidly decapitated. Brains were rapidly removed into oxygenated ice-cold Ringer’s solution (concentrations in mM: 25 NaHCO_3_, 124 NaCl, 1 KCl, 2KH_2_PO_4_, 10 glucose, 2.5 CaCl_2_, 1.3 MgCl_2_, 5 ATP, pH 7.4). Brains were cut on a vibratome into 300 μm slices in ice cold oxygenated Ringer’s solution from the most rostral aspect of the striatum to the caudal aspect of striatum as identified by the emergence of medial thalamic nuclei. Slices were equilibrated for 1 hour in oxygenated RT Ringer’s solution and then positioned in submersion recording chambers (Harvard Apparatus) on Nikon E600 IR-DIC microscopes. Slices were continuously perfused with RT Ringer’s solution (2–2.5 ml/min). D1 (eGFP-) and D2 (eGFP+) MSNs in the dorsolateral striatum were provisionally identified at the time of recordings under epifluorescence and later confirmed using confocal microscopy.

### Electrophysiology

Whole-cell patch clamp recordings were obtained from visually identified MSNs in the dorsolateral quadrant of the striatum. Electrodes were pulled on a Flaming and Brown horizontal pipette puller (model P87, Sutter Instrument) and filled with potassium methanesulfonate (KMS) internal solution, concentrations in mM as follows: (KCH_3_SO_3_ 122, MgCl_2_ 2, EGTA 5, NaHEPES 10, Na_2_ATP 5). Electrodes in Ringer’s solution had a resistance of 4–6 MΩ. Electrophysiology data was obtained using PatchMaster software (HEKA Elektronik) and EPC-9/EPC-10 amplifiers (HEKA Elektronik).

#### Assessment of intrinsic membrane and action potential properties

Passive membrane properties (resting membrane potential–Vr-, input resistance–Rn- and membrane time constant –τ- and action potential firing properties were assessed under current clamp. Vr was measured as the voltage in the absence of current injection. A series of 200 ms or 2s hyperpolarizing and depolarizing current steps was applied for the rest of the measures. The voltage responses to each step were measured at steady state and plotted on a voltage-current graph: Rn was calculated as the slope of the best-fit line through the linear portion of the plot. Membrane time constant was measured by fitting a single exponential function to the membrane voltage response to the -10 pA hyperpolarizing step. Rheobase was determined with a 10 s depolarizing current dual ramp (0–100 pA, 0–200 pA; 3.03 kHz sampling frequency). Single AP properties, including threshold and amplitude, were measured on the second evoked AP in a 200 ms current-clamp series in which the current step elicited 3 or more action potentials. An expanded timescale and the linear measure tool were used in FitMaster analysis software (HEKA Elektronik). Finally, a series of 2 s hyperpolarizing and depolarizing steps (-200 to +450 pA, using 25 or 50 pA increments, 12.5kHz sampling frequency) was used to assess repetitive AP firing. Firing rate in response to current steps was analyzed with a generalized linear model, using the genotype, MSN type, rheobase, input resistance, injected current level and their respective interactions as independent variables.

#### Assessment of spontaneous excitatory postsynaptic currents

AMPA receptor-mediated spontaneous excitatory currents (sEPSCs) were recorded for 2 min at a holding potential of -80 mV (6.67 kHz sampling frequency). Minianalysis software (Synaptosoft) was used to assess synaptic current properties including: frequency, amplitude, area, time to rise and time to decay. For assessment of kinetics, the rise and decay of averaged traces were each fit to a single-exponential function. For all synaptic current measurements, the event detection threshold was set at the maximum root mean squared noise level (5 pA).

### Streptavidin-Alexa labeling of biocytin filled neurons

Following recordings, brain slices were sandwiched between filter paper in fixative (4% paraformaldehyde) overnight at 4°C. Next, slices were washed in 0.1 M phosphate buffered saline (PBS) 3x 5 minutes. Slices were then incubated in 0.1% Tx-100/PBS for 2 h at room temperature (RT), then incubated in Streptavidin-Alexa 568 (1:500, 0.1% Tx-100/PBS) for 2d at 4°C, followed by subsequent washes in 0.1 M PBS and stored in anti-freeze solution (30% glycerol, 30% ethylene glycol in 0.05 M phosphate buffer).

### Immunohistochemistry

Following Streptavidin-Alexa staining and confirmation of D1/D2 identity with confocal microscopy, brain slices were selected for immediate coverslipping or for immunohistochemistry (IHC) based on fill quality and overall cell morphology criteria. Cells used for IHC had no dendritic varicosities and a high signal-noise ratio. IHC slices were rinsed in 0.01M PBS twice, for 10 minutes each at 4°C. Slices were then incubated in 50 mM Glycine/0.01M PBS for 1h at RT followed by two washes in 0.01M PBS at 4°C for ten minutes each. Antigen retrieval was then performed using 10 mM sodium citrate buffer in a 60–70°C water bath for twenty minutes. Brain slices were then allowed to come to RT prior to three washes in 0.01M PBS at 4°C (3x, 10 minutes each). Slices were then incubated in preblock solution (0.01M PBS, 5% bovine serum albumin (BSA), and 0.2% Tx-100) for 1h at RT. Primary antibodies (rabbit anti-Vglut1 Synaptic Systems 1:500, guinea pig anti-Vglut2 Millipore 1:1000) were diluted in an antibody diluent solution (0.1M PB, 0.2% BSAc, 0.1% Tx-100) and brain slices were incubated in primary antibody for 5 days at 4°C, with 2x 10-min incubation at 150W 40°C in a variable wattage microwave (Ted Pella, Inc), each on days 1–4. Brain slices were washed three times (10 minutes each) in 0.01 M PBS. Secondary antibodies (goat anti-rabbit Alexa-488, donkey anti-guinea pig-647, 1:200) were diluted in antibody diluent and brains slices incubated in secondary antibody solution for 3 days, with 2x 10min microwave incubations (150W, 40°C) each on days 1 and 2. On the final day, slices were washed twice in 0.01M PBS for an hour each at RT, and once in 0.1M PB for an hour to remove remaining salts. Slices were then mounted and coverslipped in Prolong Antifade (Life Technologies).

#### Immunohistochemistry controls

To validate the use of antibodies to VGLUT1 (synaptic systems) and VGLUT2 (synaptic systems), we performed two sets of control conditions. First, immunohistochemistry was performed as detailed in the above Methods section in the absence of primary antibodies (Figures a-d in S1 Fig). Second, we performed a pre-absorption assay using control protein (Synaptic Systems, Vglut1, 135-3P; Vglut2, 135-4P). Four combinations of antibodies and control proteins were incubated overnight at 4 C in 0.01 M PBS (1:3 antibody:control protein). The four combinations used to detect antibody specificity and potential cross-reactivity were: Vglut1 antibody with Vglut1 control protein, Vglut1 antibody with Vglut2 control protein, Vglut2 antibody with Vglut2 control protein, and Vglut2 antibody with Vglut1 control protein (Figures e-h in S1 Fig). The next day, 50 um brain slices were rinsed in 0.01M PBS twice, for 10 minutes each at 4°C. Slices were then incubated in 50 mM Glycine/0.01M PBS for 1h at RT followed by two washes in 0.01M PBS at 4°C for ten minutes each. Antigen retrieval was then performed using 10 mM sodium citrate buffer in a 60–70°C water bath for twenty minutes. Brain slices were then allowed to come to RT prior to three washes in 0.01M PBS at 4°C (3x, 10 minutes each). Slices were then incubated in preblock solution (0.01M PBS, 5% bovine serum albumin (BSA), and 0.2% Tx-100) for 1h at RT. Preabsorbed antibody solutions were diluted in antibody diluent solution (0.1M PB, 0.2% BSAc, 0.1% Tx-100) to their ideal concentrations (rabbit anti-Vglut1 Synaptic Systems 1:500, guinea pig anti-Vglut2 Millipore 1:1000) and brain slices were incubated in primary antibody:control protein overnight at 4°C, with 2x 5-min incubation at 150W 40°C in a variable wattage microwave (Ted Pella, Inc). Brain slices were washed three times (10 minutes each) in 0.01 M PBS. Secondary antibodies (goat anti-rabbit Alexa-488, donkey anti-guinea pig-647, 1:200) were diluted in antibody diluent and brains slices incubated in secondary antibody solution for 2 hours. Slices were washed twice in 0.01M PBS for 10 minutes each at RT, and once in 0.1M PB for ten minutes to remove remaining salts. Slices were then mounted and coverslipped in Prolong Antifade (Life Technologies) and imaged using laser scanning confocal microscopy (Lecia SPE).

### Confocal imaging

**Fig 1:**
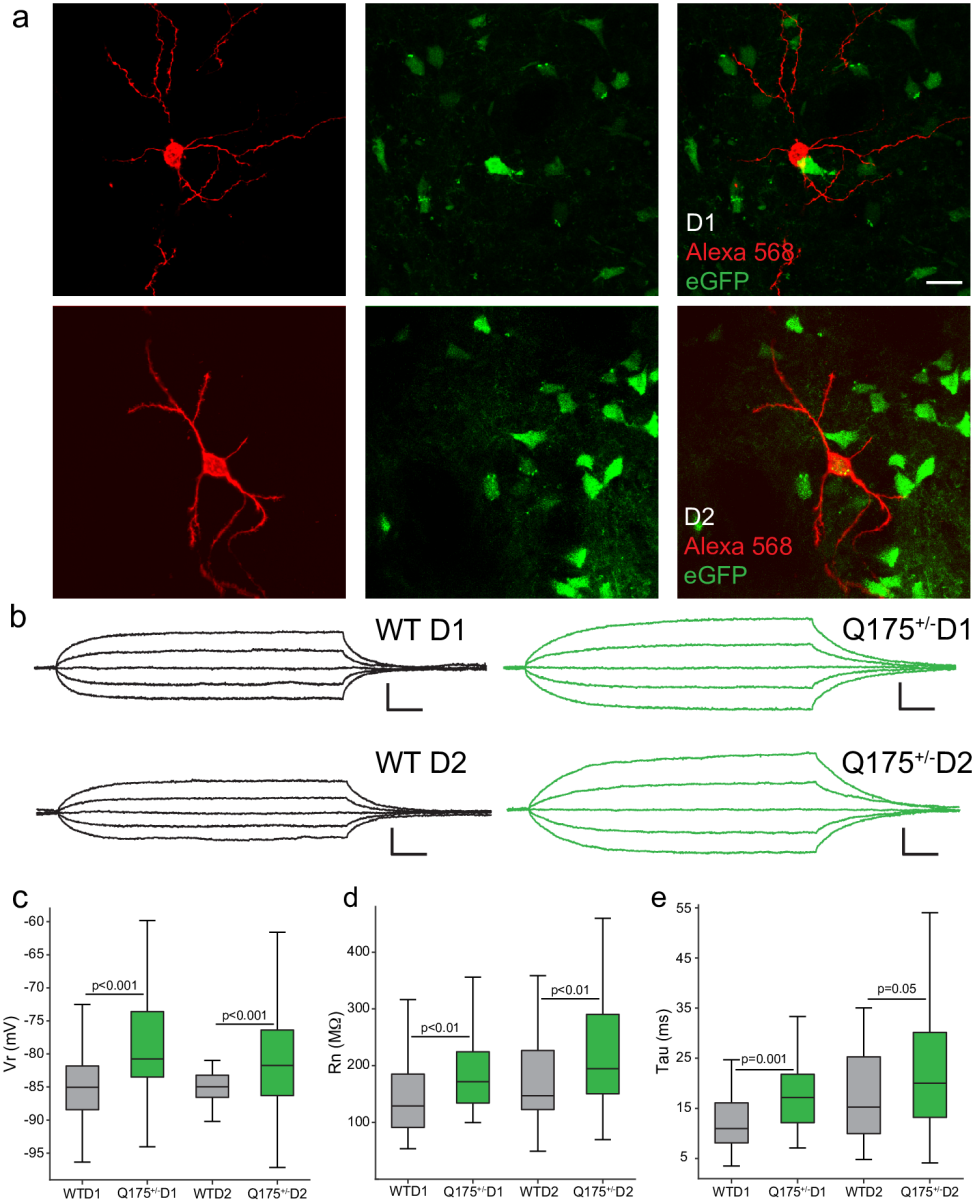
Passive electrophysiological properties of D1 and D2 MSNs are affected in 12-month-old Q175^+/-^ mice. (a) MSNs were subtyped as D1 MSNs (left panel; 568, red+/eGFP-) or D2 MSNs (right panel; 568+/eGFP+) distinguished by the expression of eGFP. (b) Voltage responses to subthreshold current steps for D1 (top) and D2 MSNs (bottom); note significantly higher input resistance in Q175^+/-^ MSNs (scale bar: 25 ms/2.5 mV). Resting membrane potential (c), input resistance (d), and the time constant, tau (e) were statistically significant between WT and Q175^+/-^ animals in both D1 and D2 MSN groups. (Boxplot solid line indicates group median and whiskers representing the 10^th^ to 90^th^ percentiles).

For verification of eGFP labeling of biocytin-filled cells, slices were placed in an inverted well slide and temporarily coverslipped for an initial imaging of the soma using a 40x oil immersion objective on a Leica SPE confocal microscope. Cell somata were scanned in their entirety in two channels, 568 and 488, to detect filled cells and the presence or absence of somal eGFP, respectively. Cells were classified as D2/eGFP+ if the 488 and 568 signals overlapped along the x-, y-, and z- planes, D1/eGFP- cells lacked eGFP signal in their soma (Fig 1a). Brain slices that contained cells met rigid criteria for morphometric analysis (zero to minimal dendritic varicosities, high signal-to-noise ratio indicative of a well filled cell, few cut branches) were either mounted in Prolong Antifade (Life Technologies) after streptavidin-alexa staining or used for immunohistochemistry. MSNs were scanned for dendritic morphometric analyses in their entirety using a Leica SPE confocal microscope with a 40x oil immersion objective obtaining a voxel size of 0.27 x 0.27 x 0.5 μm (as described previously [35,36]). Images were deconvolved with Autoquant Software and 8-bit images imported into Neurolucida 360 for reconstruction and quantitative analysis. These reconstructions are available at NeuroMorpho.Org (http://neuromorpho.org/). For assessment of spines and Vglut1 and Vglut2 appositions, 3 dendrites from each cell were chosen for imaging of spines and appositions. Each dendrite was selected by dividing the entire dendritic arbor into equal thirds and selecting the dendrite that were located perpendicular to the z-plane. In order to obtain the necessary resolution for spine sub-typing and apposition analysis, dendrites were imaged with a 63x oil emersion objective (1.4 NA) with a 2.5 zoom using a Leica SPE laser scanning confocal microscope. The resulting voxel size was 0.034 x 0.034 x 0.17 μm. Three channels were used for imaging of IHC tissue: 488 (Vglut1), 568 (filled cell), and 647 (Vglut2). Each dendrite was imaged 20 μm from the soma to exclude the aspinous region proximal to the soma. Approximately 100–120 μm of dendrite was imaged for each dendrite. Z-stacks were deconvolved using Autoquant software and 8-bit images imported into Neurolucida 360 for reconstruction, spine sub-typing, and apposition analysis.

### Densitometry

Confocal image stacks of 20 μm were acquired (63x/1.3 NA, oil-immersion; 0.056 x 0.056 x 0.5 mm voxel) from 4 fields within the dorsolateral striatum of 300 μm thick sections labeled for Vglut1 and Vglut2. All stacks were acquired at a depth of 60 μm from the top of the sections (5 wild type; 5 Q175^+/-^). In each section of the image stack, the density of Vglut1 and Vglut2 labeling in the neuropil was assessed using the particle analysis function of ImageJ (RRID:SCR_003070; [37]) and quantified as the number of labeled puncta and fractional area covered (as a fraction of the total sampled site) by labeled puncta in each confocal stack.

### Spine sub-typing and apposition analysis

Spine sub-types were classified based on spine head diameter and spine head distance from the dendritic shaft. Spines were classified as: thin (diameter ≤ 0.6 μm), mushroom (diameter > 0.6 μm), stubby (spines lacking a neck), or filopodia (length > 3 μm) [35,38]. Appositions (Vglut1+ and Vglut2+) were counted by moving step-wise through the z-plane. Fluorescent puncta were classified as appositions if they had overlapping fluorescent signal with either spines or the dendritic shaft through 3 optical slices in the z-plane (i.e. within a 1.5 frame). Appositions were quantified on the dendritic shaft, spine heads, and spine necks taking into account spine-subtype identity. These compartments were selected as MSNs are known to receive excitatory inputs on dendritic shafts and spine heads [39, 40]. Appositions to spine necks were also quantified as they were observed consistently across subjects, however, the presence of excitatory synapses to spine necks in the striatum is unclear and confirmed by 3-D structural electron microscopy. Spine and apposition quantification was performed on dendrites beginning 20 μm from the soma as there is an aspinous region of primary dendrites proximal to the soma [40].

### Statistical analyses

Statistical analyses of empirical data were performed using SigmaPlot and RStudio software. For electrophysiology comparing wild-type and Q175^+/-^ (Table 1), morphological, spine and computational analyses, unpaired *t-*tests were performed to determine statistical significance, with significance defined at *p* < 0.05. For assessing intrinsic, action potential, and synaptic properties of D1 and D2 MSNs in wild-type and Q175+/-, two-way repeated-measures ANOVAs were used to evaluate the influence of genotype and MSN type on morphology and physiology. Post hoc t-tests were performed to identify which group means differed significantly. All empirical and modeling data were tested for the assumption of normality using the Shapiro-Wilk test. Data which were not normally distributed were analyzed with the nonparametric Mann-Whitney U test.

**Table 1:** Physiological properties of unidentified MSNs.

	WT			HET			
	n	Mean	SEM	n	Mean	SEM	p-value
Vr (mV)	101	-81.51	0.91	178	-79.82	1.01	0.212
Rn (MΩ)	101	198.24	18.21	179	277.39	14.06	*<0*.*001*
Tau (ms)	93	16.52	1.13	126	23.9	1.23	*<0*.*001*
Rheobase (pA)	90	147.99	8.39	154	117.8	5.15	*0*.*002*
AP Threshold (mV)	95	-39.55	0.64	167	-37.58	0.53	*0*.*018*
AP Amplitude (mV)	95	76.73	0.99	167	71.83	0.64	*<0*.*001*
EPSC Frequency (Hz)	53	2.03	0.15	78	1.31	0.08	*<0*.*001*
EPSC Amplitude (mV)	53	13.03	0.44	76	12.89	0.34	0.804

### Computational modeling of morphologic data

All computational modeling was performed in the NEURON 7.5 simulation environment [41]. NEURON’s Import3D tool was used to convert morphologic reconstructions of the soma and dendrites from Neurolucida360 into multiple compartments on which the computational model was imposed. Imported morphologic data were checked manually and corrected as needed. After successful import, a file readable by NEURON was generated and was used in subsequent modeling. Compartment sizes were chosen automatically to be less than 1/100 of the electrotonic length constant at 100 Hz, then adjusted to produce an odd number of compartments. The time step was fixed at 0.05 ms; further reduction had no detectable impact on the simulations. All model files are available for public download on ModelDB (http://senselab.med.yale.edu/ModelDB; [42]), accession number 236310. Simulations were performed on local cluster computing resources, and remotely on the Extreme Science and Engineering Discovery Environment (XSEDE; [43]). A total of 52 neurons were reconstructed, all of which were used for modeling (n = 10, 11, 13 and 18 for WT D1, WT D2, Q175^+/-^ D1 and Q175^+/-^ D2 respectively).

The computational model included 12 active channels such as Hodgkin-Huxley fast sodium and delayed rectifier potassium in the somatic compartment, described mathematically in previous studies of MSNs of the dorsal striatum [44,45]. Parameter values for all active channels were taken from Evans et al. [45], with the persistent sodium channel parameters from Wolf et al. [44]. To explore the contribution of dendritic morphology alone to signal integration, model dendrites were passive and additional surface area due to spines were omitted. Specific membrane capacitance and axial resistivity were held fixed (*C*_*m*_ = 0.9 μF/cm^2^, *R*_*a*_ = 100 Ω·cm). Specific membrane resistance and leak reversal potential parameters for individual morphologic models were first determined by fitting model output to subthreshold current clamp data obtained empirically from the same neurons [46]. Parameters used in all models here were then set to the mean values from those fits: *R*_*m*_ = 17.3 kΩ·cm^2^ and *E*_*L*_ = -84.4 mV.

#### Electrotonic analyses

Each model neuron was analyzed with NEURON’s Electrotonic Workbench [47–49] to compute the log attenuation of voltage for transfer of signals propagating outward from the soma to the dendrites (*L*_*out*_), and inward from the dendrites toward the soma (*L*_*in*_). Both length and diameter information are incorporated into the attenuation calculations. The mean outward and inward attenuations, ${\overset{-}{L}}_{out}\;$ and ${\overset{-}{L}}_{in}$ respectively, were computed by averaging the attenuation measure over all traversed paths in the dendrites as previously described [50–52]. These attenuation measures are dependent on the frequency of the input signal [53]. Since action potentials and EPSCs can be decomposed into signals at frequencies ranging from 0 Hz to 500 Hz [50], inward and outward attenuation measures were computed over the same range.

#### EPSC modeling

Excitatory synaptic events activations were modeled as AMPA receptor-gated events using NEURON’s Exp2Syn, NetCon and NetStim mechanisms as in Amatrudo et at. [51], with a reversal potential of 0 mV. Each synapse was modeled as a double exponential, activated as an identical random Poisson process with a mean number of activations per second (λ). Model synapse parameters were fit for 12 neurons that had empirical data for spine counts and EPSC recordings (3 per group). In each model synapses were inserted uniformly along the dendrites, totaling ~20% of the spine numbers measured empirically. Holding the soma at -70 mV under voltage clamp and activating each synapse once, the rise and decay time constants and the maximum conductance of the AMPA receptor-gated channels were adjusted to fit the mean EPSC statistics of each neuron. The parameter λ was then adjusted so that the simulated EPSC frequency at the soma over a 50 sec time interval equaled the empirically-determined mean EPSC frequency.

To quantify whether dendritic morphology affected synaptic attenuation differently in Q175^+/-^ D1 versus WT D1 MSNs, another series of simulations activated excitatory synapses throughout the dendrites. Synapses were inserted throughout the dendrites of one representative WT D1 MSN (100 each in the proximal, medial, and distal regions), then the corresponding synapse density was computed. The same synapse density was then applied to all D1 MSN models (10 WT D1, 13 Q175^+/-^ D1). Each synapse was activated once at 50 ms intervals using identical synaptic kinetics (*g*_*AMPA*_ = 0.21 nS, *t*_*rise*_ = 2.29 ms, *t*_*decay*_ = 1.80 ms, equal to the mean WT D1 parameters fit above). The somatic EPSC amplitude versus synapse distance from the soma was recorded. A multiple regression was used to fit the natural logarithm of EPSC amplitude vs. genotype, MSN type, and traversed distance from the soma, resulting in an exponential fit of EPSC amplitude vs. the dependent variables.

## Results

### Unidentified MSNs from 12-month-old Q175^+/-^ mice exhibit altered electrophysiological properties

In order to compare our findings to previous studies of ‘unidentified’ MSNs, we grouped D1 and D2 MSNs within WT and within Q175^+/-^ genotypes (Table 1). Q175^+/-^ MSNs exhibited a significantly higher input resistance and lower rheobase compared to WT (p = 0.0006 and p = 0.0024 respectively). Action potential threshold was depolarized and amplitude reduced in Q175^+/-^ compared to WT MSNs (p = 0.018 and p = 0.00005 respectively). Finally, the frequency of EPSCs was significantly reduced in Q175^+/-^ compared to WT MSNs (p = 0.00006), while EPSC amplitude and kinetics did not differ. These data replicate those in previous reports of physiological changes to unidentified MSNs in Q175^+/-^ compared to WT mice [27,28].

### Intrinsic membrane properties of D1 and D2 MSNs in 12-month-old Q175^+/-^ vs. WT mice

**Fig 2:**
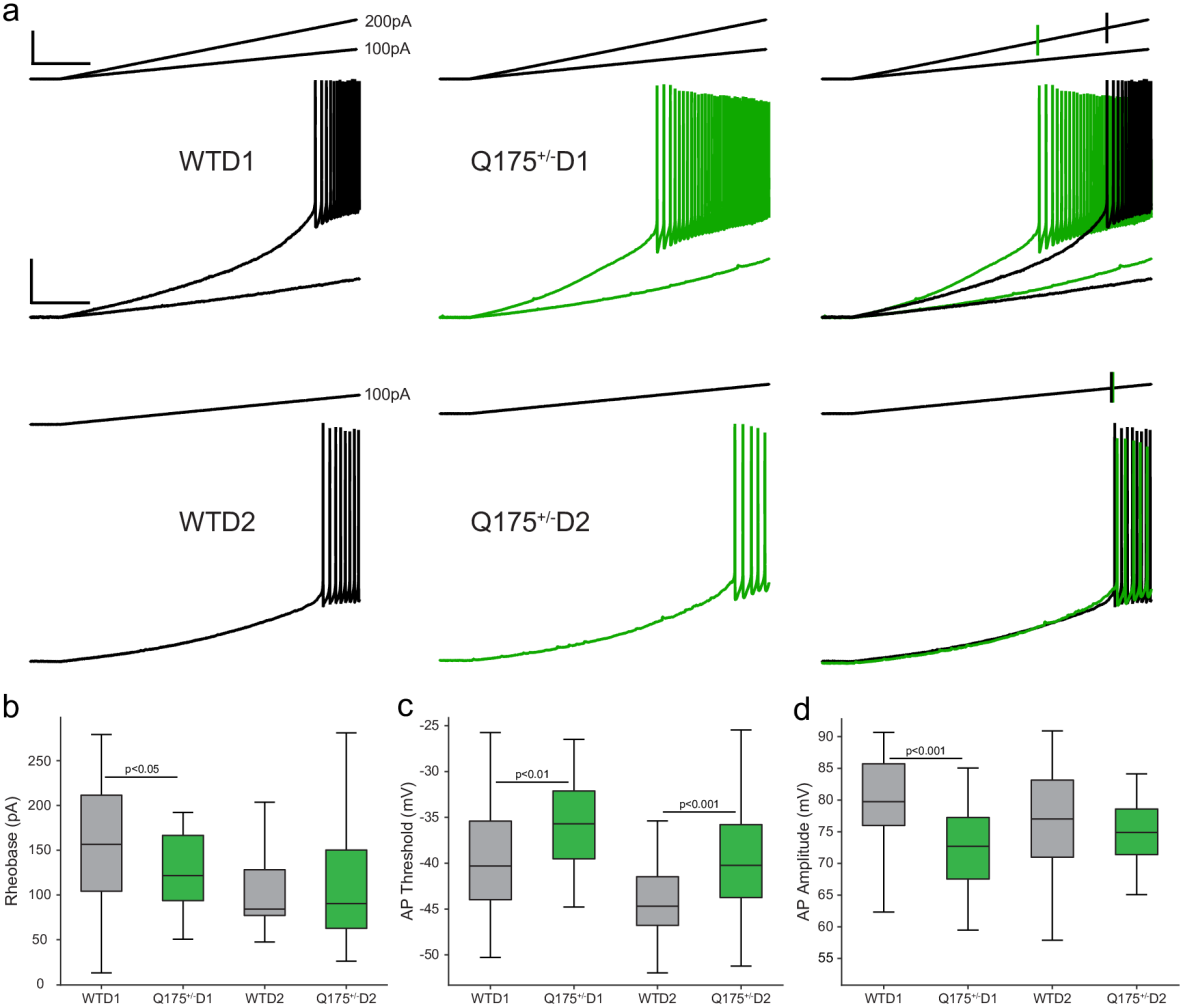
Action potential firing properties of D1 and D2 MSNs. (a) Voltage responses (lower traces; scale bar: 20mV/1s) to current ramps (upper traces; scale bar: 100pA/1s) used to determine rheobase (WT left, Q175^+/-^ center; overlay right, vertical lines on right-most current ramps indicate rheobase). (b) Rheobase was significantly reduced in D1, but not D2 MSNs from Q175^+/-^ mice. (c) Action potential threshold was significantly depolarized in both Q175^+/-^ D1 and D2 MSNs. (d) Action potential amplitude was significantly reduced in Q175^+/-^ D1 but not D2 MSNs.

MSNs were classified as D1 or D2 types using confocal microscopy to confirm the presence (D2, eGFP+) or absence (D1, eGFP-) of eGFP. Normative electrophysiological properties of D1 vs. D2 MSNs in WT mice are provided in S1 Table. As previously reported [54,55] both rheobase and action potential threshold were significantly higher in D1 than in D2 MSNs, however Rn and Vr did not differ between the subtypes. There was a statistically significant interaction between genotype and cell type affecting resting membrane potential (Vr), input resistance (Rn), and time constant, tau (τ) (Fig 1b–1e, Vr: F_(2, 191)_ = 11.022, p < 0.0001; Rn: F_(2, 185)_ = 14.097, p < 0.0001; τ: F_(2, 189)_ = 14.130, p < 0.0001). Both D1 (eGFP-) and D2 (eGFP+) MSNs in Q175^+/-^ mice exhibited a significantly depolarized Vr, higher Rn, and longer time constant compared to WT neurons (p-values for post hoc t-tests given in Table 2). Rheobase–the minimal current required to elicit an action potential–was affected by a significant interaction between genotype and MSN type (Fig 2a and 2b; F_(2, 173)_ = 8.159, p < 0.0001). Rheobase was significantly reduced in D1 MSNs in Q175+/- compared to WT D1 MSNs, while Q175+/- D2 MSNs did not differ from WT D2 MSNs (p-values for post hoc t-tests in Table 2). Two-way ANOVA showed a significant interaction between genotype and cell type affecting action potential threshold and amplitude (Fig 2c, AP threshold F_(2, 189)_ = 17.869, p < 0.0001; Fig 2d, AP Amplitude F_(2, 185)_ = 12.755, p < 0.0001). Action potential threshold was lower for both D1 and D2 MSNs in Q175^+/-^ compared to WT mice, while action potential amplitude was reduced in D1 but not D2 MSNs from Q175^+/-^ mice (post-hoc t-test p-values in Table 2). Action potential rise time, fall time, and the duration of the AP at half-amplitude were affected by a significant interaction between genotype and cell type (Rise: F_(2, 181)_ = 6.141, p = 0.003; Fall: F_(2,176)_ = 3.602, p = 0.029, Duration at half F_(2, 181)_ = 6.374, p = 0.002). While AP rise time was significantly longer in Q175^+/-^ D1 MSNs (but not D2; post-hoc p-values in Table 2), fall time did not differ between wild-type and Q175^+/-^ groups, rather, fall time differed between Q175+/- D1 and Q175+/- D2 cells (data not shown). The duration of the action potential at half amplitude was longer in Q175^+/-^ D2-MSNs (post-hoc p-value in Table 2). Firing rate in response to depolarizing 2-sec current steps was analyzed with a generalized linear model (R^2^ = 0.61). Rheobase contributed significantly to this relationship (*p* < 0.001), but genotype, MSN type, input resistance, and any of their interactions did not (*p* > 0.23 for all remaining terms). As expected there was a strong negative correlation between firing rate (at 180 pA, R^2^ = 0.55) and rheobase (data not shown).

**Table 2:** Physiological properties of D1 and D2 MSNs from WT vs. Q175+/- mice.

	D1						
	WT			Q175+/-			
	n	Mean	SEM	n	Mean	SEM	p-value
Vr (mV)	40	-85.42	0.80	43	-79.19	1.23	*<0*.*001*
Rn (MΩ)	42	144.28	10.08	37	186.89	10.81	*0*.*005*
Tau (ms)	41	12.46	0.97	36	18.18	1.33	*0*.*001*
Rheobase (pA)	40	156.94	10.92	34	127.45	6.90	*0*.*024*
AP Threshold (mV)	42	-39.15	0.98	40	-35.56	0.74	*0*.*004*
AP Amplitude (mV)	41	79.86	1.17	40	72.43	1.11	*<0*.*001*
EPSC Frequency (Hz)	28	2.15	0.20	15	1.68	0.12	*0*.*044*
EPSC Amplitude (mV)	29	11.99	0.50	16	11.54	0.57	0.550
	D2						
	WT			Q175+/-			
	n	Mean	SEM	n	Mean	SEM	p-value
Vr (mV)	22	-85.18	0.56	89	-81.42	0.83	*<0*.*001*
Rn (MΩ)	27	172.38	16.27	82	226.52	11.26	*0*.*008*
Tau (ms)	27	17.53	2.06	88	22.34	1.27	*0*.*049*
Rheobase (pA)	23	104.63	9.27	79	107.22	6.92	0.821
AP Threshold (mV)	26	-44.04	1.00	84	-39.39	0.65	*<0*.*001*
AP Amplitude (mV)	27	76.48	1.91	80	75.02	0.53	0.461
EPSC Frequency (Hz)	20	1.81	0.19	51	1.44	0.08	0.077
EPSC Amplitude (mV)	20	13.69	0.70	50	12.96	0.40	0.366

### Reduced frequency of sEPSCs in D1 but not D2 MSNs in 12-month-old Q175^+/-^ mice

**Fig 3:**
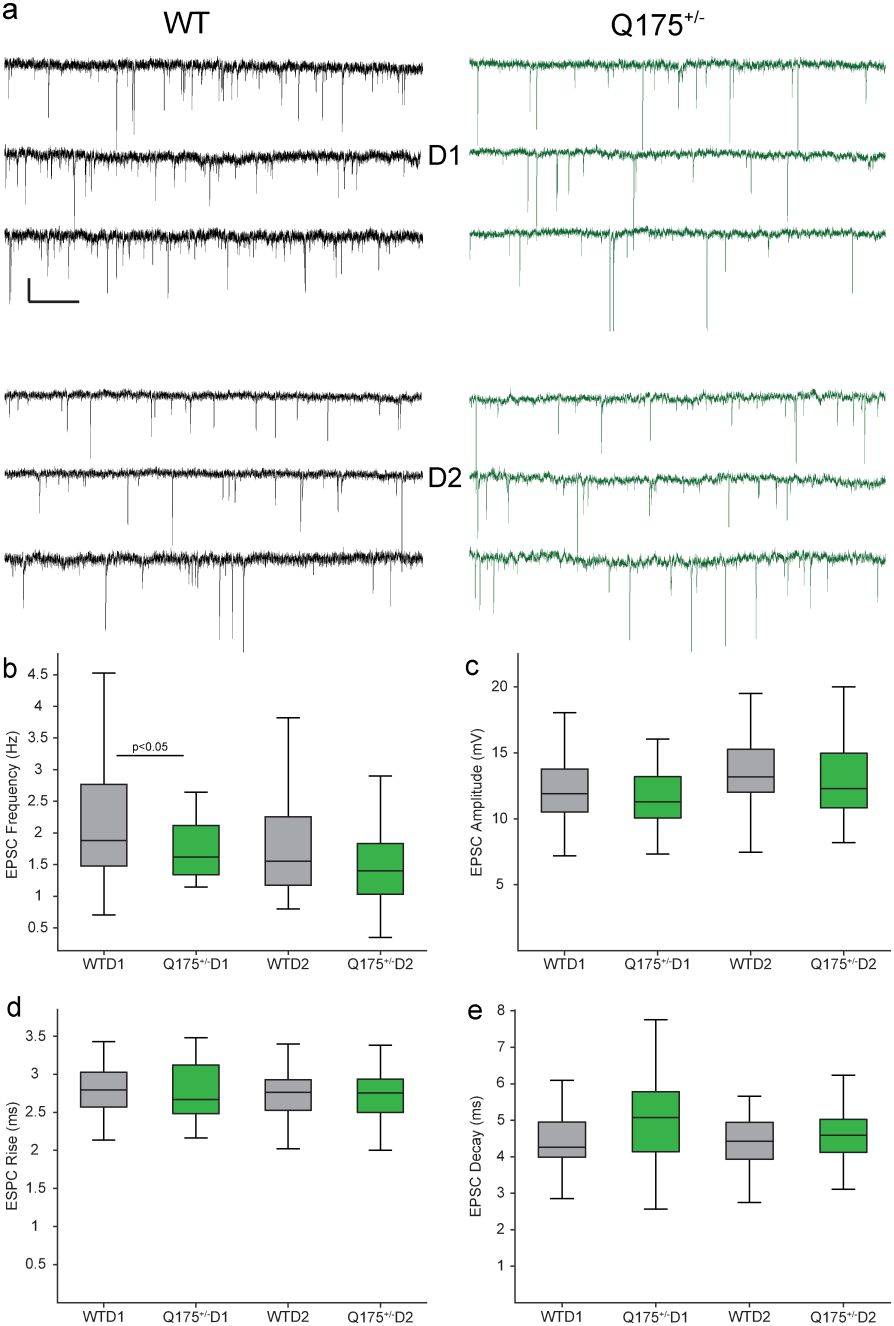
Spontaneous excitatory postsynaptic currents are less frequent in 12-month-old Q175^+/-^ D1 MSNs. (a) Representative traces of sEPSCs from D1 and D2 MSNs (scale bar: 10pA/1s). (b) EPSC frequency was significantly lower in Q175^+/-^ D1 but not D2 MSNs compared to WT. (c-e) EPSC amplitude, rise and fall times did not differ between Q175^+/-^ and WT MSNs.

Spontaneous excitatory post-synaptic currents were measured in WT and Q175+/- D1 and D2 MSNs. There was a significant interaction between genotype and cell type affecting the frequency (Hz) of sEPSCs (Fig 3, F_(2,126)_ = 8.297, p < 0.0001), however, amplitude, rise and decay times showed no such interaction (data not shown). Q175^+/-^ D1 MSNs exhibited a reduced mean frequency of sEPSCs, while sEPSC frequency did not differ between D2 MSNs of WT and Q175+/- mice. These changes to excitatory synaptic currents, along with alterations in intrinsic membrane and action potential properties, suggest that the expression of mHTT in the Q175^+/-^ affects D1 MSN electrophysiological function to a greater extent than D2 at 12 months of age. As physiological response properties are heavily influenced by morphological features, we sought to determine if the structural properties of subtype-specific MSNs were altered in Q175^+/-^ mice.

### Dichotomous changes to dendritic topology of 12-month-old Q175^+/-^ D1 vs. D2 MSNs

**Fig 4:**
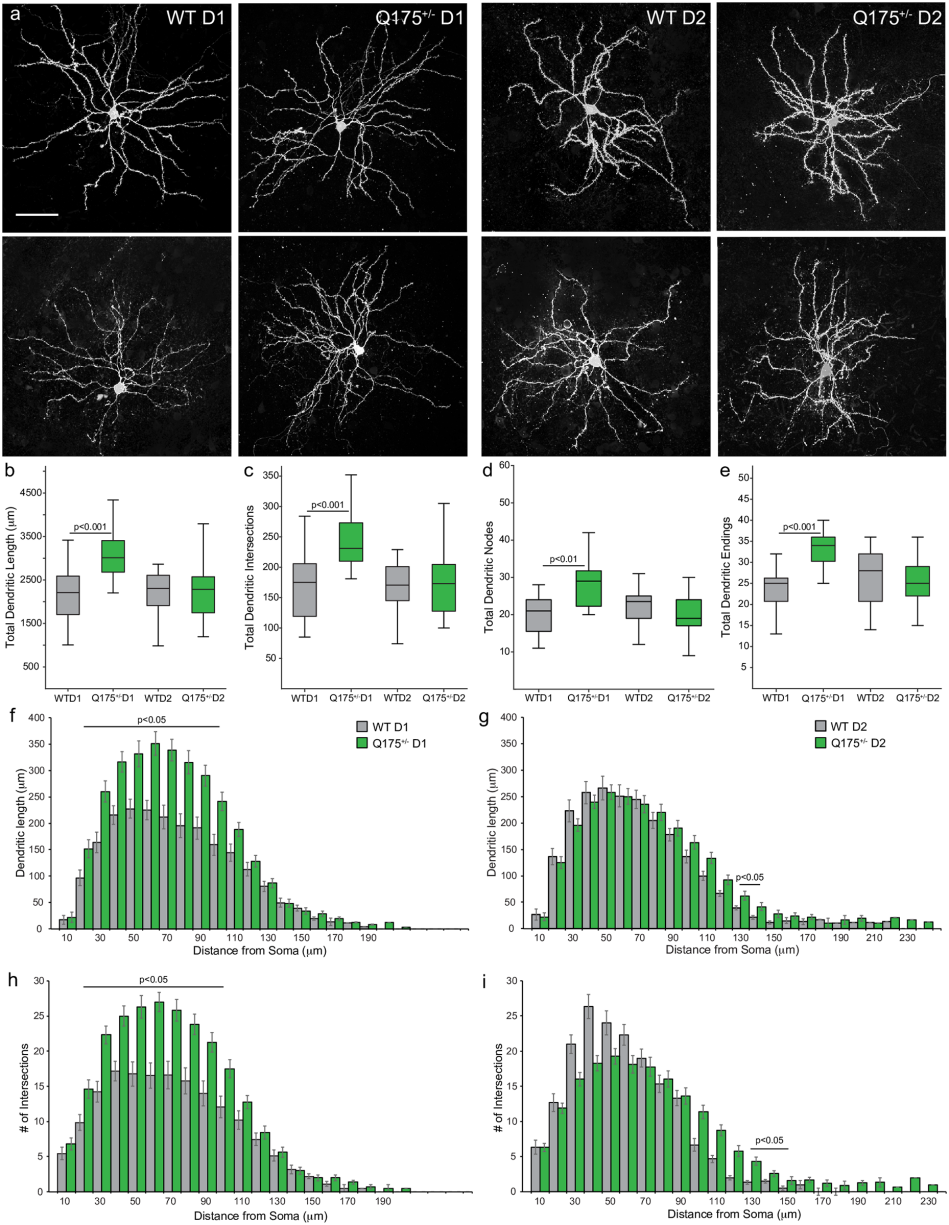
Dendritic topology is altered in 12-month-old Q175^+/-^ D1 MSNs. (a) Confocal images of representative MSNs (scale bar: 100μm). (b-d) Q175^+/-^ D1 MSNs exhibit greater total dendritic length (b), total dendritic intersections (c), total number of nodes, (d) and total dendritic endings (e), indicative of an increase in arbor complexity. (f, g) Sholl plots showing increased dendritic length in Q175^+/-^ D1 but not D2 MSNs relative to WT. (h, i) Q175^+/-^ D1 but not D2 MSNs exhibit a higher number of dendritic intersections than WT.

MSN morphology was assessed using high resolution confocal microscopy and subsequent reconstruction of the cell soma and dendritic arbor (Fig 4a). Normative morphological properties of D1 vs. D2 MSNs in WT mice did not differ (S1 Table); interestingly, this finding (from 12-month old mice) is in agreement with a previous report of no difference in the dendritic topology of D1 and D2 MSNs in adult mice [40] but differs from a previous report of greater dendritic length and complexity of D1 compared to D2 MSNs in juvenile mice [55]. The volume and surface area of MSN somata did not differ across genotypes (data not shown). Total dendritic length, the total number of dendritic intersections, the total number of dendritic branching points (nodes), and the number of dendritic endings (termination of a given branch which there are no further branch points) were all affected by a significant interaction between genotype and cell type (length: F_(2,67)_ = 12.117, p < 0.0001; intersections: Fig 4c, F_(2,67)_ = 12.195, p < 0.0001; nodes: Fig 4d, F_(2,65)_ = 6.428, p < 0.001; endings: Fig 4e, F_(2,64)_ = 12.711, p<0.0001). Post-hoc analyses showed that Q175^+/-^ D1 but not D2 MSNs exhibited significantly greater dendritic arbor length and complexity compared to WT. Q175^+/-^ D1 MSNs possessed a significantly greater mean total dendritic length (Fig 4b), number of dendritic intersections (Fig 4c), number of nodes (Fig 4d) and number of dendritic endings (Fig 4e) compared to WT D1 MSNs, while Q175^+/-^ D2 MSNs did not differ from WT. Sholl analysis of dendritic arbors revealed that Q175^+/-^ D1 have a greater dendritic length and dendritic intersections 20–100 μm from the soma (Fig 4f and 4h). While the mean dendritic parameters did not differ between WT and Q175^+/-^ D2 MSNs (Fig 4b–4e), Sholl analysis revealed a modest increase in the dendritic length and number of intersections in the distal most compartments (130–150 μm; Fig 4g and 4i).

### Thin spines are selectively lost in D1 MSNs of 12-month-old Q175^+/-^ mice

**Fig 5:**
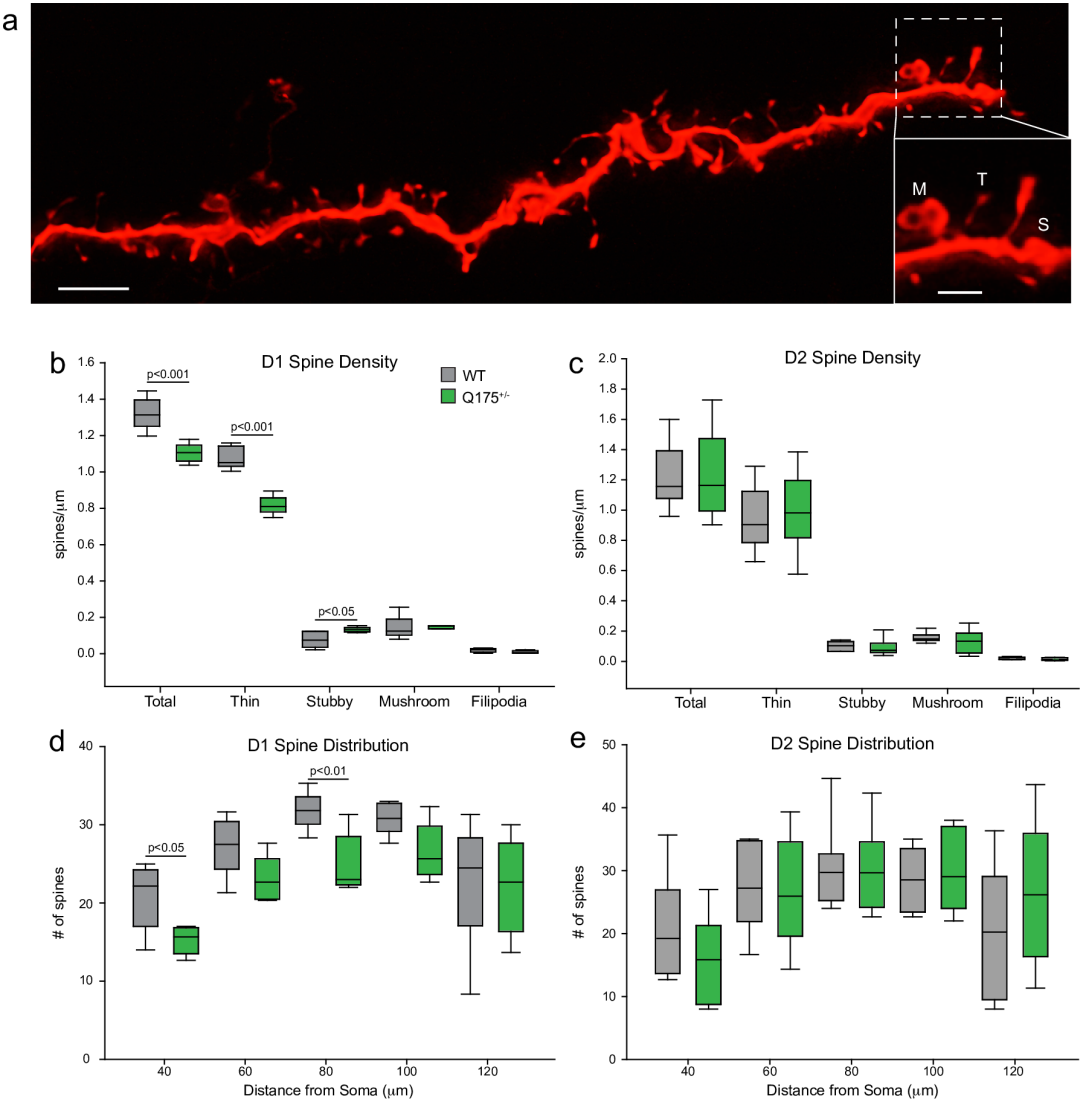
Selective loss of thin spines in 12-month-old Q175^+/-^ D1 MSNs. (a) Confocal images of a dendritic branch with spines from a WT MSN (scale bar: 3μm); inset shows spine subtypes- M- mushroom; T- thin; S: stubby (Scale bar: 1μm). (b, c) Plots showing spine density on D1 and D2 MSNs. There was a significant reduction in total and in thin spine density in Q175^+/-^ D1 but not D2 MSNs. (d, e). Spine distribution is modestly altered as a function of distance from soma in D1 and unchanged in D2 Q175^+/-^ MSNs. (Boxplot solid line indicates group median and whiskers representing the 10^th^ to 90^th^ percentiles; crosses, outliers).

To assess the distribution and density of spines–the major recipients of excitatory inputs to MSNs- high resolution 63x confocal scans of individual dendritic branches were analyzed (Fig 5a). For each cell (WT D1, n = 6; WT D2, n = 6; Q175^+/-^ D1, n = 6; Q175^+/-^, n = 6) the dendritic arbor was divided into thirds and a single dendrite imaged from each section. Dendrites that were chosen had no dendritic varicosities and were perpendicular to the z-plane to allow for maximal imaging of spines and appositions. The total density of spines was significantly reduced on Q175^+/-^ D1 MSN dendrites (Fig 5b; p < 0.001) while total spine density of D2 MSNs did not differ between genotypes (Fig 5c). When individual spine sub-types were assessed separately (Fig 5a, inset), Q175^+/-^ D1 MSNs had fewer thin spines compared to WT D1 MSNs and a greater density of stubby spines while mushroom and filopodia subtypes were comparable between genotypes (Fig 5b; thin, p < 0.001; stubby, p < 0.05). We then assessed spine number within the dendritic compartment closer to the soma (20–120 μm from the soma) as this region exhibited the greatest changes in dendritic complexity in Q175^+/-^ D1 MSNs. Sholl analysis showed that Q175^+/-^ dendrites had fewer spines within this compartment (Fig 5d). Thin spine number in this compartment was also reduced consistent with the total spine population in Q175^+/-^ D1 MSNs (data not shown).

### Glutamatergic inputs to the striatum and appositions onto filled D1- and D2-MSN dendrites and spines

**Fig 6:**
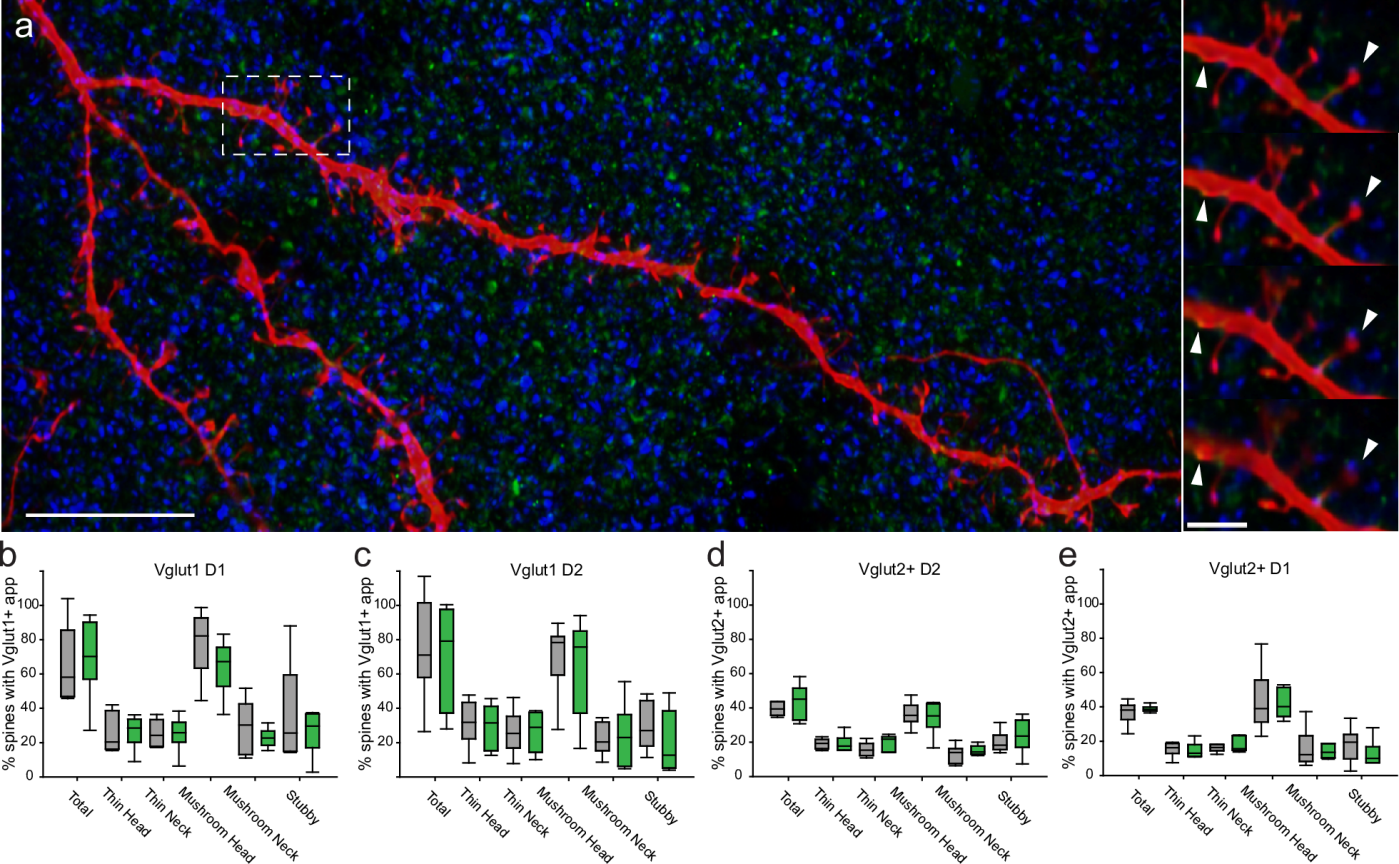
Glutamatergic appositions onto identified D1 and D2 MSNs do not differ between WT and Q175^+/-^ groups. (a) Confocal images of MSN dendrites and spines (red, Alexa 568) with glutamatergic appositions (Vglut1, cortical, green; Vglut2, subcortical- principally thalamic-, blue; Scale bar: 10μm). Puncta are apposed to the dendritic shaft as well as to spines (right panels; Scale bar: 2.5μm; white arrows indicate Vglut1 and Vglut2 appositions followed through confocal image stacks in series). (b, c) The proportion of spines with Vglut1 appositions on D1 and D2 MSNs did not differ between Q175^+/-^ and WT groups. (d, e) The proportion of spines with Vglut2 appositions on D1 and D2 MSN dendrites did not differ between Q175^+/-^ and WT groups.

MSNs receive excitatory inputs primarily from the neocortex and thalamus; inputs which can be differentiated by immunohistochemical labeling of vesicular glutamate transporter sub-types-Vglut1 and Vglut2 respectively (Fig 6a). We first assessed the density of Vglut1 and Vglut2 label within the dorsolateral striatum with particle analysis. Particle analysis showed no difference in optical density of either label when comparing WT and Q175+/- animals (data not shown). Since assessment was performed using confocal microscopy, Vglut1 (green) and Vglut2 (blue) puncta that overlapped with dendritic shaft or spines (over 3 optical slices) of biocytin-filled MSNs were considered “putative synapses” or appositions since only electron microscopy can validate true synapses. Appositions were quantified on compartments that have been shown to be recipients of excitatory input on MSNs (e.g. dendritic shaft and spine heads; [39,40]) as well as spine necks (Fig 6a–6e). Apposition density over the entire length of the dendritic shaft was comparable between WT and Q175^+/-^ D1 and D2 MSNs for both Vglut1 and Vglut2 appositions (mean ± SEM, units: appositions/mm; Vglut1: WTD1 = 0.27±0.11, Q175+/-D1 = 0.20±0.05, WTD2 = 0.32±0.12, Q175+/-D2 = 0.23±0.07; Vglut2: WTD1 = 0.16±0.05, Q175+/-D1 = 0.15±0.09, WTD2 = 0.19±0.06, Q175+/-D2 = 0.15±0.03). The ratio of Vglut1 to Vglut2 was consistently 1.5:1 for both D1 and D2 MSNs in both WT and Q175^+/-^ groups. Assessment of apposition types were compared to the corresponding spine sub-type (i.e. % of mushroom head spines with either Vglut1 or Vglut2 appositions) revealed no differences between WT and Q175^+/-^ (Fig 6b–6e).

### Modeling the impact of structural changes on signaling properties

**Fig 7:**
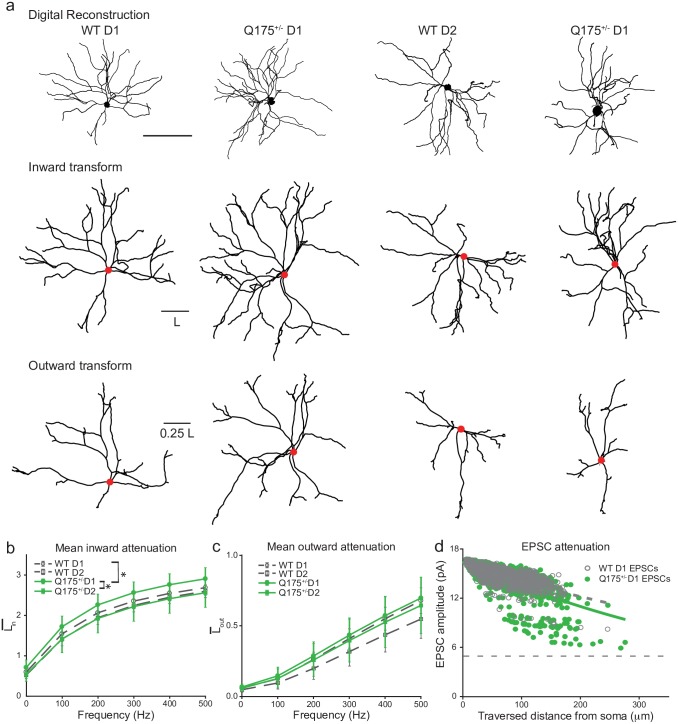
Simulated incoming signals attenuate more in Q175^+/-^ D1 than WT D1 MSNs. (a) Morphologic reconstructions (top), plus inward (middle) and outward (bottom) morphoelectrotonic transforms of representative neurons from each group (Scale bar: 100μm). (b-c). Mean inward (b) and outward (c) attenuation across all the dendrites among all four groups (*n* = 53 total). Data from WT MSNs are shown in gray with open; Q175^+/-^ MSNs are shown in green with closed symbols. Symbols are circles for D1, squares for D2. Data from D1 MSNs are shown in solid lines and D2 in dashed lines. Groups that differ significantly are marked with (*). (d**).** Simulated EPSCs attenuate more in Q175^+/-^ D1 MSNs than WT D1. Shown is EPSC amplitude vs. synaptic distance from the soma in 9 WT D1 and 14 Q175^+/-^ D1 model neurons. Lines show the best-fit exponential decay vs. distance of each group. Horizontal dashed line indicates 5 pA, the minimum EPSC amplitude detected in empirical data.

Our past studies showed that morphologic differences can lead to changes in dendritic signal attenuation [50–52,56]. Since there were significant changes in the morphology of MSNs in Q175^+/-^ animals, we applied our prior electrotonic analysis methods to these neurons. We used 3D morphologic reconstructions to construct compartmental models from 53 neurons among the four groups and computed the mean inward attenuation from the dendritic tips to the soma (${\overset{-}{L}}_{in}$), and mean voltage attenuation outward from the soma to the tips (${\overset{-}{L}}_{out}$), for oscillatory inputs at various frequencies. Our previous work shows that ${\overset{-}{L}}_{in}$ and ${\overset{-}{L}}_{out}$ are good predictors of simulated EPSC amplitudes and AP backpropagation respectively. Fig 7a shows morphoelectrotonic transforms of a total of four MSNs, one from each group. These transforms scale the length of each anatomical branch to illustrate how much voltage attenuates in response to input (here, at 500 Hz): a longer branch implies greater attenuation. The digital reconstructions (top row) can be compared to the transforms representing inward attenuation toward the soma (*L*_*in*_, middle) and outward attenuation away from the soma (*L*_*out*_, bottom).

The mean inward attenuation ${\overset{-}{L}}_{in}$ of WT D1 and WT D2 neurons did not differ, nor did mean outward attenuation ${\overset{-}{L}}_{out}$ at low frequencies, however at higher frequencies there was a significantly greater ${\overset{-}{L}}_{out}$ in WT D2 versus WT D1 MSNs (Fig 7c; p < 0.035 for 300–500 Hz). Beyond these normative comparisons, ${\overset{-}{L}}_{in}$ was significantly greater in Q175^+/-^ versus WT D1 MSNs for high input frequencies (Fig 7b; *p* < 0.045 for 400–500 Hz), and greater in D1 versus D2 of Q175^+/-^ mice at all frequencies (*p* < 0.015). These results predict that morphologic changes in Q175^+/-^ D1 MSNs will impact the attenuation of input signals along the dendritic trees propagating from the dendrites to the soma. In contrast, there was no evidence that mean outward attenuation ${\overset{-}{L}}_{out}$ differed in WT vs. Q175^+/-^ for either D1 or D2 neurons (Fig 7c; *p* > 0.11 for all comparisons).

To demonstrate the functional relevance of the ${\overset{-}{L}}_{in}$ results, we activated individual excitatory synapses (AMPA-mediated) distributed at a uniform density throughout the dendrites in models of 23 D1 MSNs (n = 9 for WT, n = 14 for Q175^+/-^). We held the somas under voltage clamp and recorded the resulting somatic EPSCs with identical synaptic parameters for all model MSNs. Fig 7d shows the inverse relationship between somatic EPSC amplitude and distance of the activated synapses along the dendrites. The space constant parameter of an exponential fit was larger in Q175^+/-^ D1 MSNs compared to WT D1, indicating that EPSCs from synapses in the distal dendrites attenuated more in Q175^+/-^ D1 MSNs (R^2^ = 0.537, p < 0.001). None of the simulated EPSCs in WTD1 or Q175^+/-^ D1 MSNs fell below the 5pA threshold used for detecting synaptic events empirically. Therefore, these results suggest that the increased attenuation does not account for the ~20% reduced EPSC frequency of Q175^+/-^ MSNs compared to WT D1.

**Fig 8:**
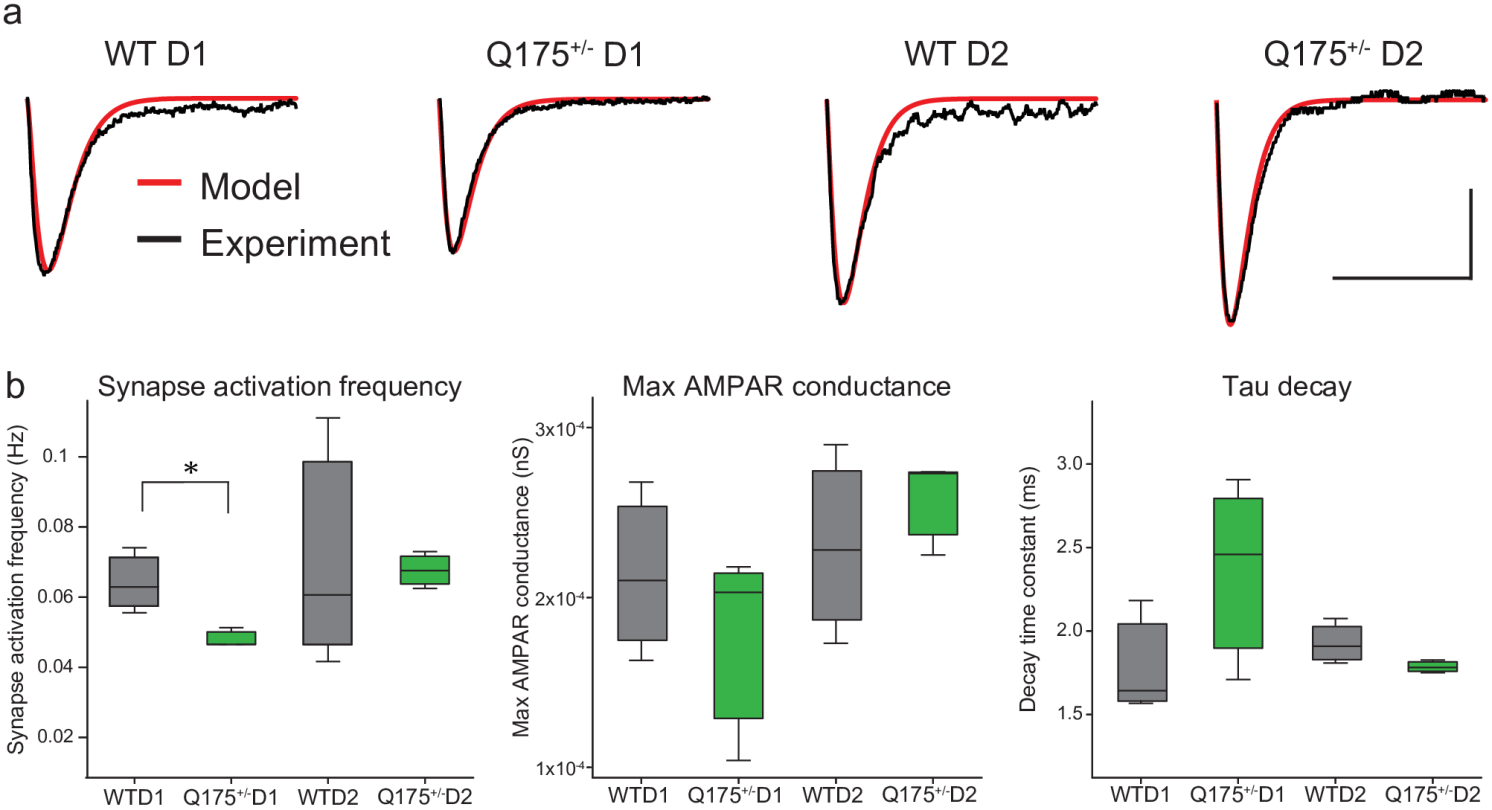
The frequency of synaptic events is lower in model Q175^+/-^ D1 neurons. (a) Mean sEPSC traces. For one neuron in each group (WT D1, Q175^+/-^ D1, WT D2 and Q175^+/-^ D2), we simulated the AMPAR-mediated sEPSCs. Empirical data shown in black, model in red. (b) The bar and whiskers plots of optimized AMPAR-mediated sEPSC model parameters fit to empirical data (3 per group). The synaptic activation frequency was lower in Q175^+/-^ D1 models compared to WT D1 (*), but otherwise fitted parameters did not differ.

To further examine why EPSC frequencies might differ in Q175^+/-^ D1 vs. WT D1 neurons, we optimized parameters controlling the conductance and kinetics of AMPA receptor-gated channels to fit the mean model EPSCs to empirical data (Fig 8a and 8b; *n* = 3 models per group). The mean conductance and kinetics of AMPA receptor-gated channels did not differ in any Q175^+/-^ vs. WT models (p > 0.05 for all comparisons). The mean activation frequency of individual excitatory synapses was significantly lower in Q175^+/-^ D1 compared to WT D1 models (T-test, p = 0.041), but did not differ in Q175^+/-^ D2 vs. WT D2 models (T-test, p = 0.74). These modeling predictions imply that presynaptic changes may contribute to the observed reduction in EPSC frequency of Q175^+/-^ D1 vs. WT D1 mice.

## Discussion

Striatal MSNs are highly vulnerable to dysfunction and degeneration in Huntington’s disease and have consequently been the focus of extensive study in numerous mouse models of the disease. In the present study, we replicated previous reports of significantly increased input resistance, lower rheobase, lower action potential amplitude and threshold, and reduced frequency of sEPSCs in unidentified MSNs in the Q175^+/-^ knock-in model [27–29] and other HD models [24–26]. In studies of human HD brain, findings of selective loss of D2-MSN projections with relative sparing of D1-MSN projections early in the course of the disease and subsequent loss of D1 projections [16–21] has led to the wide-spread view that D2 MSNs are selectively vulnerable to degenerative changes–particularly early in the disease course. These sequential changes in indirect and direct pathway projections to target structures are consistent with the pathological motor phenotypes seen early (chorea) and late (bradykinesia) in HD. While evidence for differential progressive loss of efferent projections from the striatum is robust, there is conflicting evidence that the D1 and D2 neurons that give rise to these projections undergo sequential degeneration. For example, Joyce et al. [57] used quantitative autoradiography to demonstrate a 65% reduction of D1 receptors and a much lesser 28% loss of D2 receptors in HD striatum. Several PET studies of manifest HD patients found no difference in the degree of reduction of striatal D1 versus D2 binding [58,59]. Meta-analysis of more recent longitudinal PET studies [22,23] indicate that in the striatum of premanifest HD patients D2 receptor binding declines at a rate of ~4–6% annually and that D1 receptor binding declines at a rate of ~2%, while these rates are 3% and 5% respectively in manifest HD patients [22,23]. Thus, while D2 receptors (though not necessarily the neurons themselves) in the striatum are reduced at premanifest stages of the disease, at manifest stages both D1 and D2 receptors and MSNs are lost.

Direct assessment of the mechanisms underlying the potential selective vulnerability of D1 vs D2 MSNs is not possible in human HD patients and so must be undertaken in animal models of the disease. Few studies to date have examined selective vulnerability of MSNs in models of HD, though there have been reports of differential changes to excitatory:inhibitory synaptic balance in- [31] and corticostriatal inputs to- the two populations [32]. Thus, Andre et al. [31] have reported that mean EPSC frequency is increased in D1 MSNs in the YAC128 and BACHD models at 1.5 months of age and reduced at 12 months. By contrast, EPSC frequency did not differ between HD model and WT D2 MSNs at either age. Furthermore, corticostriatal inputs to D1 MSNs are lost in 1-year old Q140 model mice while these inputs to D1-negative neurons (presumptive D2 neurons) are spared [32]. Recently, Barry et al. [60] showed that D1 and D2 output pathways are differentially affected in two different mouse models of HD with reduced amplitude of inhibitory synaptic inputs to the substantia nigra and increased duration of inhibitory synaptic inputs to the external segment of the globus pallidus. These studies are consistent with the idea that D1 and D2 MSNs are differentially affected by mHTT in mouse models just as they seem to be in human HD. However, it remains to be determined whether the temporal and qualitative nature of differences between MSN subtypes in the human are recapitulated in mouse models.

We assessed whether there is selective vulnerability of D1 vs D2 MSNs in 12-month-old Q175^+/-^ x D2-eGFP mice because this heterozygous knock-in (KI) model has WT/mHTT allele ratio construct validity and exhibits dysfunctional motor phenotypes reminiscent of the human disease. Detailed assessment of the structure and function of identified MSNs revealed differential changes to D1 and D2 neurons in the striatum of 12-month-old Q175^+/-^ compared to WT mice. Thus, while the passive membrane properties of both D1 and D2 Q175^+/-^ neurons differed from WT, with regard to all other electrophysiological and structural properties assessed, D1 MSNs exhibited significantly more robust and extensive changes than did D2 MSNs at this age.

### Electrophysiological changes are more prominent in D1 than in D2 MSNs

The passive membrane properties of both D1 and D2 MSNs from Q175^+/-^ mice differed from those of WT mice, with increased input resistance, more depolarized resting membrane potential and longer time constant. The increased input resistance was not due to reductions in cell soma size since there was no difference in soma size of Q175^+/-^ and WT MSNs. Though not directly assessed here, it is possible that the changes in passive properties that appear to be ubiquitous in MSNs across models of HD may be due to alterations in ionic conductances, as has previously been proposed. Indeed, reductions in both inwardly and outwardly rectifying potassium conductances in MSNs in the R6/2, Tg- CAG100, and, most recently, the Q175 mouse models of HD have been described [61–64]. Furthermore, full striatal proteome analysis of Q175^+/+^ and Q175^+/-^ has revealed significant reductions in Kv2.1, Kv4.2, KChIP2, Kir2.1, Kir 2.3 and SLACK potassium channels [65]. Future studies are required to determine the functional relevance of MSN potassium channel changes in the Q175^+/-^ model. By contrast to passive membrane properties, only D1 neurons exhibited reduced rheobase and reduced action potential amplitude- two other hallmark features reported in unidentified MSNs in HD model mice. Furthermore, a significantly reduced frequency of glutamatergic sEPSCs was only observed in the D1 population. Previous studies of unidentified MSNs in the Q175^+/-^ model have consistently reported similar significant reductions in the frequency of sEPSCs [27–29]. Indersmitten et al. [28] suggest that the reduction in frequency may be attributed to a loss of postsynaptic substrate (dendritic spines) which they attributed to reductions in afferent synaptic inputs from the cortex and thalamus. While these investigators found evidence for reduced spine density on MSNs consistent with this idea, there is currently no direct anatomical evidence for changes in glutamatergic inputs to MSNs in the Q175^+/-^ model.

### D1 but not D2 MSNs exhibit marked morphological changes and reduced sEPSC frequency

We ascertained whether selective changes to the electrophysiological features of D1 but not D2 neurons were due to differential dystrophic changes using detailed quantification of dendritic topology and spine densities of individual identified neurons. In a previous report, the dendritic morphology of unidentified MSNs did not differ in the Q175^+/-^ or Q175^+/+^ mouse models of HD compared to WT at 2-, 7- or 12-months of age [28]. Here, we observed a proliferative expansion (increased length and number of intersections) of the dendritic arbors of D1 neurons, while D2 neurons were unchanged in this same model at 12-months of age. Whether this marked increase in the dendritic arbor length and complexity of D1 neurons represents a compensatory reaction to upstream changes in the striatal synaptic circuitry or is a change inherent to the neurons themselves remains an interesting question for future studies.

Given that the frequency of sEPSCs was significantly reduced in Q175^+/-^ D1 (but not D2) MSNs, we quantified the distribution of dendritic spines on the neurons using high resolution confocal microscopy. D1 but not D2 MSNs from Q175^+/-^ mice exhibited a significant reduction in the density of spines, particularly in proximal regions where proliferative dendritic changes were also observed. Thin spines were specifically lost, while the density of the more stable mushroom spines was unaltered. This finding is consistent with the notion that in 12-month-old Q175^+/-^ mice D1 MSNs undergo reductions in the most plastic dendritic spines and that these changes may result in a lower frequency of synaptic currents in these neurons. Furthermore, the larger density of stubby spines on Q175^+/-^ D1 MSNs may be a failure of stubby spines to develop into thin spines or may represent the retraction of thin spines.

Whether the changes seen in D1 MSNs represent a compensatory response to altered inputs or a pathological loss of function in Q175^+/-^ mice remains to be determined. The hyperexcitability of Q175^+/-^ D1 MSNs, for example, could be a compensatory response for the reduced frequency of excitatory inputs and the increased attenuation of remaining synaptic currents due to increased dendritic filtering. Likewise, the reduction in spine density might compensate for the increased dendrite length and branching in D1 MSNs as an attempt to maintain a similar number of spines overall. In such cases, the lack of plasticity might imply greater mHTT toxicity in D2 MSNs. This would agree with a recent study [66] in which synaptic plasticity was impaired in D2 but not D1 MSNs of BACHD and Q175^+/-^ mice, due to impaired signaling of tyrosine-related kinase B (TrkB) receptors. On the other hand, the observed changes in Q175^+/-^ D1 MSNs themselves might represent a greater toxic effect in this sub-type, leading to direct pathway signaling impairment and motor deficits as discussed below. Our findings highlight the importance of studying identified MSNs, since grouping neurons into a single (heterogeneous) population does not allow for identification of sub-type specific differences and likely obscures differences between HD model and WT groups. Notably, previous findings in human post-mortem tissue showed a large variability in changes to dendritic and spine topography in HD; both abnormal sprouting and degeneration of MSN dendrites and spines have been described [67]. The potential for MSN morphologic instability due to mHTT dependent mechanisms, may result in multiple diverse phenotypic changes such as those seen in HD postmortem tissue.

### Vglut1+ and Vglut2+ appositions onto D1 and D2 MSN spines and dendrites are unaltered

To determine whether reduced sEPSC frequency was associated with alterations in afferent glutamatergic inputs to the dorsolateral striatum in general and to neurons from which recordings were obtained specifically, we employed immunocytochemical quantification of Vglut1+ (corticostriatal) and Vglut2+ (principally thalamostriatal) appositions onto spines and dendritic shafts of filled neurons. Assessment of Vglut1 and Vglut2 immunoreactive puncta in the dorsolateral striatum using densitometry revealed that there was no change in the levels of these presynaptic terminals in Q175^+/-^ compared to WT mice (not shown). Furthermore, there were no significant changes in the number, density or distribution of immunoreactive excitatory appositions onto the dendritic shafts or spines of either D1 or D2 Q175^+/-^ MSNs compared to WT. Thus, it is unlikely that the functional changes in synaptic frequency seen in the neurons from which recordings were obtained are directly due to alterations in afferent inputs *per se*. This negative finding differs markedly from that of Deng et al. [32], who reported a 63% reduction in Vglut1+ terminals synapsing on D1+ spines and a 10% reduction of these terminals onto D1- (presumptive D2 spines) in the striatal neuropil of Q140 mice. It is also inconsistent with the ~40% reduction in Vglut2+ terminals adjacent to both D1+ and D1- spines seen in that study [32]. The highly discrepant findings between the present study and Deng et al., may be due to the different mouse models examined and/or to the very different methodological approaches in the two studies (semi-quantitative immuno-electron microscopy in neuropil vs. confocal immunocytochemical assessment of punctae on processes of individual identified neurons).

### Functional implications of structural changes to D1 MSNs

We used computational modeling to quantify the functional implications that might arise from the structural changes observed in Q175^+/-^ MSNs. To isolate how differences in dendritic morphology affected signal propagation, model dendrites were passive. Our modeling predicts only minor differences in the attenuation of signals in WTD1 vs. WTD2 neurons. This result differs somewhat from a past study that modeled EPSCs and backpropagation in D1 and D2 MSNs [55], but can be explained by the morphologic differences in the juvenile mice analyzed there versus the 12-month old mice here. Our work suggests that the more complex arbors of D1 Q175^+/-^ neurons has little to no effect on the backpropagation of action potentials outward from the soma. However, we predict that the increased complexity in Q175^+/-^ D1 MSNs does lead to greater dendritic filtering and attenuation of signals propagating to the soma from the dendrites. The lower spine density in proximal dendrites of Q175^+/-^ D1 vs WTD1 neurons and altered thin and stubby spine densities were not modeled here, but these spine changes would be insufficient to compensate for the added surface area due to increased complexity of Q175^+/-^ D1 MSNs. The attenuation may be enhanced or compensated by dysregulation of voltage- and calcium-gated ion channels that may occur in HD as mentioned above [61–63,65]; for example, dendritic hyperexcitability has been associated with Kv4.2 depletion in a mouse model of Alzheimer’s Disease [68]. Future modeling work to predict intrinsic mechanisms that underlie firing properties in each of these four groups will help elucidate interactions between morphology and ion channel dysregulation in MSNs in mouse models of HD. Such findings will complement recent modeling studies of the effects of HD on network-level function in the striatal microcircuit [69,70].

Together, our results suggest that reduced sEPSC frequency seen in D1 but not D2 neurons, is influenced by changes in dendritic architecture and spine density in D1 but not D2 neurons. That said, modeling predicts that it is unlikely that the dendritic changes alone account for the reduced sEPSC frequency observed here and by others [27–29]. Furthermore, reduced frequency of glutamatergic events cannot be accounted for by reduced afferent input in the neuronal population that we assessed, since no change was found. However, while the anatomical inputs appear to be intact, there is likely a reduction in presynaptic release probability *per se*, as demonstrated in a previous report of reduced miniature EPSC frequency in Q175^+/-^ MSNs [27]. Additional evidence in support of the latter idea is provided by our synaptic modeling which predicts a lower frequency of individual excitatory synaptic events in Q175^+/-^ vs. WT D1 neurons but no change in kinetics or in D2 MSNs. Furthermore, empirical studies show that mHTT disrupts endosomal compartments and calcium dynamics, actions which could profoundly alter the number of neurotransmitter vesicles available to dock and release neurotransmitter [71,72]. How might reduced synaptic excitation influence the direct (D1) pathway’s role in normal motor function? As noted above, if reduced synaptic excitation reduces dysfunctional effects of hyperexcitability seen in HD this effect could contribute to the maintenance of normal function. However, Q175^+/-^ mice have been reported to exhibit motor deficits in the rotarod task as early as 30 weeks of age [33]. Nakamura et al. [73] demonstrated that performance on the rotarod test was specifically impaired in D1R knock out (KO) but not D2R KO mice, suggesting that the D1 MSN direct pathway plays a key role in motor regulation of fine motor control and voluntary movement. Thus, it is plausible that reduced sEPSC frequency in D1, but not D2 MSNs could underlie reduction in D1 MSN output and subsequently impaired rotarod performance observed in these mice.

### Conclusions and future directions

The preponderance of studies of human HD brain support the idea that D2 MSNs are preferentially impacted during the early manifest stages of disease and that at later stages both D1 and D2 neurons are equally affected. In the advanced age symptomatic Q175^+/-^ mice, we thus expected that significant pathological alterations would be observed in both D1 and D2 neurons. Surprisingly, our data provides strong evidence that D1 MSNs are more selectively altered while D2 MSNs are–with the exception of alterations to passive membrane properties and AP threshold–relatively less changed in these mice at 12-months of age. This leads to the obvious question of whether D2 neurons are in fact spared in this model throughout the lifespan or whether the absence of observable changes in D2 neurons was a consequence of the experimental design and model. It is possible that in young Q175^+/-^ mice, D2 neurons are in fact dystrophic but that by 12 months of age those D2 neurons most impacted by the presence of mHTT either have died or are too unhealthy to record from successfully in *in vitro* slices. However, had this been the case we should have encountered more difficulty recording from Q175^+/-^ D2 than from WT D2 neurons and this was not the case. Another possibility is that the presence of eGFP–which is known to be neurotoxic in itself–[74–76], altered the structure and function of both WT and Q175^+/-^ neurons artificially, thus obscuring any potential changes due to the presence of mHTT in the Q175^+/-^ mice. However, while technically this is a possibility, we do not believe this to be the case since the properties of D2 neurons showed no evidence of cytotoxicity such as has been described in association with eGFP [74–76]. Finally, it is possible that D2 neurons are altered in ways not assessed in the present study; for example, they may undergo alterations in axonal projections and/or integration into the complex striatal and extra-striatal microcircuitry. Future detailed physiological and anatomical studies of identified MSNs in young Q175^+/-^ mice and of Q175^+/-^ x D1-eGFP mice will provide insight into these possibilities. Nevertheless, D1 neurons showed substantial changes that provide further insight into potential neurotoxic effects of mHTT and suggest selective responsivity of D1 MSNs to mHTT. The Q175^+/-^ model is increasingly employed as a prominent preclinical model of HD and as such these findings provide important new avenues for investigation into selective vulnerability of neurons to mHTT.

## Supporting information

S1 Fig Immunohistochemical validation of Vglut1 and Vglut2 antibodies. Images of immunohistochemical controls for Vglut1 and Vglut2 shown in the absence (a, Vglut1; c, Vglut2) or presence (b, Vglut1; d, Vglut2) of primary antibody. Antibody specificity was validated by antibody reabsorption with control protein to Vglut1 and Vglut2 (e, Vglut1 antibody, Vglut1 control protein; f, Vglut1 antibody, Vglut2 control protein; g, Vglut2 antibody, Vglut2 control protein; h, Vglut2 antibody, Vglut1 control protein).
(TIF)

S1 Table Physiological and morphological properties of WT D1 vs. WT D2 MSNs. (DOCX)
