# Supplementary figures and images for: Differential changes to D1 and D2 medium spiny neurons in the 12-month-old Q175+/- mouse model of Huntington’s Disease
Source: PLoS One. 2018 Aug 17;13(8):e0200626. doi: 10.1371/journal.pone.0200626 (PMC6097649; doi:10.1371/journal.pone.0200626)

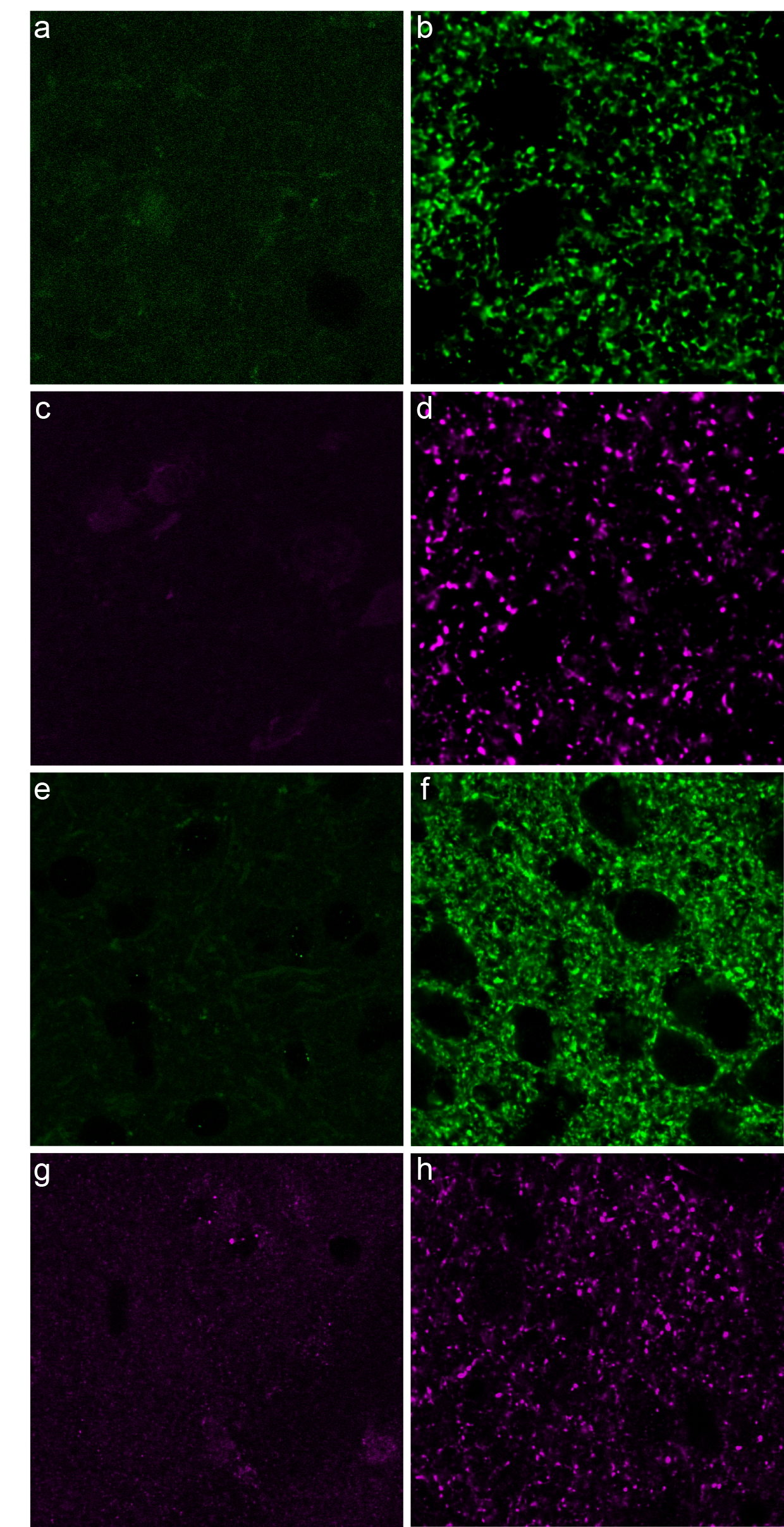

Supplement: S1 Fig — Images of immunohistochemical controls for Vglut1 and Vglut2 shown in the absence (a, Vglut1; c, Vglut2) or presence (b, Vglut1; d, Vglut2) of primary antibody. Antibody specificity was validated by antibody reabsorption with control protein to Vglut1 and Vglut2 (e, Vglut1 antibody, Vglut1 control protein; f, Vglut1 antibody, Vglut2 control protein; g, Vglut2 antibody, Vglut2 control protein; h, Vglut2 antibody, Vglut1 control protein). (TIF) [file pone.0200626.s001.tif]
