# Supplementary material for: Differential changes to D1 and D2 medium spiny neurons in the 12-month-old Q175+/- mouse model of Huntington’s Disease
Source: PLoS One. 2018 Aug 17;13(8):e0200626. doi: 10.1371/journal.pone.0200626 (PMC6097649; doi:10.1371/journal.pone.0200626)
Supplement: S1 Table — (DOCX) [file pone.0200626.s002.docx]

|  | D1 | | | D2 | | |  |
| --- | --- | --- | --- | --- | --- | --- | --- |
|  | n | Mean | SEM | n | Mean | SEM | p-value |
| Physiology: |  | | |  | | |  |
| Vr (mV) | 40 | -85.42 | 0.80 | 22 | -85.18 | 0.56 | 0.80 |
| Rn (MΩ) | 42 | 144.28 | 10.08 | 27 | 172.38 | 2.06 | 0.14 |
| Tau (ms) | 41 | 12.46 | 0.97 | 27 | 17.53 | 2.06 | *0.03* |
| Rheobase (pA) | 40 | 156.94 | 10.92 | 23 | 104.63 | 9.27 | *<0.01* |
| AP Threshold (mV) | 42 | -39.15 | 0.98 | 26 | -44.03 | 1.00 | <0.001 |
| AP Amplitude (mV) | 41 | 79.86 | 1.17 | 27 | 76.48 | 1.91 | 0.13 |
| EPSC Frequency (Hz) | 28 | 2.15 | 0.20 | 20 | 1.81 | 0.19 | 0.20 |
| EPSC Amplitude (mV) | 29 | 11.99 | 0.50 | 20 | 13.69 | 0.70 | 0.43 |
| Morphology: |  | | |  | | |  |
| Dendritic Length (μm) | 13 | 2142.80 | 197.89 | 14 | 2160.29 | 164.08 | 0.94 |
| Dendritic Intersections | 13 | 167.23 | 17.98 | 14 | 166.50 | 12.89 | 0.97 |
| Dendritic Nodes | 13 | 20.08 | 1.63 | 14 | 23.29 | 2.12 | 0.22 |
| Dendritic Endings | 13 | 23.69 | 1.60 | 13 | 25.54 | 2.00 | 0.46 |
| Spine Density (spines/μm): |  | | |  | | |  |
| Total | 6 | 1.32 | 0.04 | 6 | 1.22 | 0.10 | 0.33 |
| Thin | 6 | 1.07 | 0.03 | 6 | 0.94 | 0.10 | 0.20 |
| Mushroom | 6 | 0.14 | 0.03 | 6 | 0.16 | 0.01 | 0.65 |
| Stubby | 6 | 0.08 | 0.02 | 6 | 0.10 | 0.01 | 0.28 |
| Filipodia | 6 | 0.02 | 0.00 | 6 | 0.02 | 0.00 | 0.88 |
